# Using Agent-Based Models to Develop Public Policy about Food Behaviours: Future Directions and Recommendations

**DOI:** 10.1155/2017/5742629

**Published:** 2017-03-21

**Authors:** Philippe J. Giabbanelli, Rik Crutzen

**Affiliations:** ^1^Department of Computer Science, Northern Illinois University, DeKalb, IL, USA; ^2^Department of Health Promotion, CAPHRI, Maastricht University, Maastricht, Netherlands

## Abstract

Most adults are overweight or obese in many western countries. Several population-level interventions on the physical, economical, political, or sociocultural environment have thus attempted to achieve a healthier weight. These interventions have involved different weight-related behaviours, such as food behaviours. Agent-based models (ABMs) have the potential to help policymakers evaluate food behaviour interventions from a systems perspective. However, fully realizing this potential involves a complex procedure starting with obtaining and analyzing data to populate the model and eventually identifying more efficient cross-sectoral policies. Current procedures for ABMs of food behaviours are mostly rooted in one technique, often ignore the food environment beyond home and work, and underutilize rich datasets. In this paper, we address some of these limitations to better support policymakers through two contributions. First, via a scoping review, we highlight readily available datasets and techniques to deal with these limitations independently. Second, we propose a three steps' process to tackle all limitations together and discuss its use to develop future models for food behaviours. We acknowledge that this integrated process is a leap forward in ABMs. However, this long-term objective is well-worth addressing as it can generate robust findings to effectively inform the design of food behaviour interventions.

## 1. Introduction

Many countries are faced with a very high prevalence of overweight and obesity. In the United States, 75% of adult men and 67% of adult women are overweight or obese [1]. Similarly, most adults are overweight in Canada [2] and the United Kingdom [3]. In addition, current models developed for Canada, Australia, or the United States predict continued increase in the rates of obesity [2, 4, 5]. Hence, overweight and obesity is a key area for public health. In particular, new policy documents have emphasized the role of eating patterns in achieving healthy weight. For example, the UK Department of Health stated that “increasing physical activity is important but, for most of us who are overweight and obese, eating and drinking less is key to weight loss” [3]. In line with this emphasis, our paper is focused on food behaviours, that is, the food-related decisions that individuals make under the influence of the physical, economical, political, or sociocultural environment.

Intervening on food behaviours through these different sources of influence requires complex intervention. Several organizations, such as the UK Medical Research Council, have emphasized that modelling could be a valuable tool when developing and evaluating such complex interventions [6]. Indeed, modelling has been used to test policy scenarios (also known as “what-if” questions) or understand the interconnectedness of factors driving food behaviours. Modelling approaches can be broadly divided into three categories [7]: qualitative aggregate models, quantitative aggregate models (also called* macrosimulation*), and quantitative individual models (also called* microsimulation*). In this review, we are interested in the last category and specifically* agent-based models* (ABM). (While this review focuses on ABM, other quantitative individual modelling techniques are sometimes used interchangeably with ABM and may thus benefit from the resources articulated here. In particular, “network models” as they are used in obesity research can sometimes be simplified ABMs, where researchers detailed peer interactions but simplified (existing) sources of environmental influences.) This modelling technique can capture the decision-making processes of individuals and how they interact both with others and with their environment. ABMs have demonstrated that they could be useful when it comes to studying food behaviours. For example, they have improved our understanding of the role that social influences play in weight dynamics [8–11]. However, current procedures to develop and use these models suffer from three limitations.

First, ABMs have been lacking in details regarding the role that* both* peers and the food environment play [12], despite the fact that “the relationships of single categories of potential determinants [of food behaviours] can only be valuable if they are studied in their interplay” [13]. The assumption was often made that only the food environment around the home or work environment would impact diet [14–16]; that is, models tend to represent only what surrounds the home or work location (e.g., using a buffer area). We will refer to this assumption as the “proximity hypothesis.” While this hypothesis has been prevalent in studies on individual food exposure [17], it has led to inconsistent findings [18] suggesting that people are not primarily using what is geographically proximate [19]. This is because individuals navigate a multiplicity of environments [20] (e.g., purchase food while commuting) and may even shop far away from home/work in the case of low-income groups, which are disproportionally facing obesity [14, 21].

A second and related limitation is the fact that developing detailed ABMs of food behaviours requires the ability to fully utilize large amounts of data on how individuals navigate the food environment. Indeed, using such rich datasets allow us to develop, calibrate, and validate models that go beyond the proximity hypothesis. However, the acquisition, cleaning, and analysis of detailed spatial datasets of food behaviours have typically been done in a separate strand of literature (primarily geography [22]) from the ones on developing ABMs of food behaviours (typically public health nutrition and obesity research [23]). Finally, policies can tackle several determinants of food behaviours at the same time, in ways that depend on a local constituency's characteristics and agenda. Current practices are to test one policy at a time using an ABM, thus missing possible synergies between interventions. In other words, ABMs can be used to take a systems approach and realize that the sum of interventions is greater than the individual components, but such use of ABMs is not yet the norm.

While several reviews have been dedicated to modelling in obesity (including ABMs for food behaviours) [24–26], they generally focus on how models have been used and what initial steps could be taken by public health researchers to use modelling. In contrast, this paper seeks to support the future development of models addressing the limitations identified above. It does so by (i) reviewing how existing methods and datasets can be used to address each limitation, and (ii) proposing a way to combine these resources. The directions and recommendations in this paper can be used to develop the next generation of models, going beyond the proximity hypothesis and integrating the interrelated sources of influence at work in food behaviours. Using such an integrated (or systems science) perspective, we will be able to develop more policy-relevant models that allow searching for synergistic combinations of interventions while paying close attention to possibly detrimental side-effects.

As we seek to support a systems science approach to ABMs for food behaviours, our review integrates resources across disciplines. Thus, rather than a systematic review on one aspect, our scoping review organizes selected resources along the natural progression of model building. Our methods section starts by explaining how to link food exposure and utilization: we review how this is done in current models and we detail how performing data mining on datasets generated by the Global Positioning System (“GPS” datasets) can improve on this current situation. Then, having assumed that we can partly understand the complex interrelationships between food exposure and utilization, we review how virtual platforms can be developed to estimate how our current utilization of the food environment would change in reaction to new policies. Our methods conclude by examining how such virtual platforms can contribute to identifying synergistic combinations of interventions targeting the political, sociocultural, economic, and physical food environments to promote healthier eating practices across different socioeconomic groups. Then, the implications of such an integrated procedure are discussed, with particular emphasis on expected benefits and challenges given current practices.

## 2. Methods

### 2.1. Linking Food Exposure and Utilization of the Food Environment

#### 2.1.1. Overview

The few ABMs developed with a focus on the food environment followed the proximity hypothesis [14–16]. The ability of an ABM to be highly detailed points out that the reliance on the proximity hypothesis was not dictated by methodological constraints. The lack of familiarity of public health researchers with ABM may historically have contributed to models being designed by primarily technical teams without being aware of other hypotheses. However, ABM has been widely communicated together with other modelling techniques to public health researchers in the recent years, through initiatives including thematic issues of prominent journals in the field [32, 33]. Even recent models developed by interdisciplinary teams rely on the proximity hypothesis, suggesting that the limitations are neither methodological nor a lack of awareness of food behaviours. Rather, there have been two key limitations: a lack of data that precisely captures how individuals navigate the food environment and the ability to derive an insight from this data. In the next two sections, we address how we can go beyond the proximity hypothesis using existing techniques and datasets.

#### 2.1.2. Identifying and Processing the Right Datasets

A review found that the lack of data on individual mobility patterns and the limited attention that it received in the literature were key knowledge gaps in the development of ABMs [34]. This problem has been partly addressed in recent models of physical activity but remains salient in modelling food behaviours. For example, highly individual-centric ABMs have recently been developed for physical activity [35] and were used to assess inequalities [36] through detailed survey data, but food decisions and routines were modelled as being random (e.g., places were selected uniformly at random). Going further requires highly detailed data tracking human mobility patterns, preferentially using the Global Positioning System (GPS).

There exists a plethora of GPS datasets. However, these datasets need to have a sufficient large time window in order to be representative of the individuals' behaviour. A dataset collected over a single day, such as in [37], may be insufficient to infer behavioural patterns. In addition, in order to calibrate and validate a system with reasonable confidence margins, there is a need to have “enough” participants. While this is highly dependent on what aspects of food decision-making a model seeks to capture, datasets having only 37 participants [38] are not sufficient for modelling purposes. Several studies have been completed in the last years on the food environment using GPS, and they have both a sufficient time window and over a hundred individuals. A sample of such studies is provided in Table 1, and a dedicated 2016 review details the use of GPS dataset for the food environment [22].

In addition, new datasets have been collected that contain an even larger number of individuals, with a mean wear time for the GPS of 1 week: the University of Cambridge's Fenland Study (http://www.mrc-epid.cam.ac.uk/research/studies/fenland) (*n* = 805) and the University of Washington's Seattle Obesity Study 2 (http://depts.washington.edu/uwcphn/work/cor/diet_disparities_SOSII.shtml) (SOS2; *n* = 493). Some of these datasets also provide travel diaries. For example, in SOS2, participants completed a diary entry for each trip. This asked about where/when the trip started/ended and what activity occurred (snack/meal/beverage/food shopping). Such diaries provide a “ground truth” for how individuals interact with their food environment, which can otherwise only be inferred from the GPS wearing. For example, signal can be temporarily lost when individuals go inside a food venue (e.g., restaurant in a mall, canteen in an office building), and we could only hypothesize which venue(s) may have been visited. In the absence of “ground truth” such inference may still be valuable, although that would depend on the design of the built environment: one could confidently categorize a signal disappearing in front of a restaurant and reappearing one hour later as “eating at this location,” but if signal disappears in a shopping mall then the error margin about supposed activities is much larger. Other datasets may provide dietary measures (Table 1), which summarize a participant's diet and/or shopping patterns. Dietary measures are particularly useful as they can be used to double check what can be inferred from the GPS. Through these datasets and their metadata, we are thus now in the position where we have high quality data to model food behaviours, and a data science pipeline needs to be put in place accordingly.

The raw signals (known as “GPS traces”) from selected datasets need to be transformed into “GPS trips” via a cleaning process before the data can be analyzed or used within an ABM. Research in transportation has been particularly active in developing cleaning methods for GPS [39]. Even if their purpose was eventually to analyze travel behaviour rather than food behaviour, these cleaning methods apply in our case. The GPS trace first has to undergo noise filtering, which is necessary in order to address jumps between points (e.g., when signal was temporarily lost because of either indoor activities or real GPS device problems). While some methods remove zero-speed points generated when the user does not move in order to generate trips, they can be useful depending on the context [40]. To develop ABMs of food behaviour, we* would* keep these points as they can be used to identify that a user stopped at a food venue. As part of the cleaning process, incorrect positions should be removed and map-matching algorithms should be used to assign positions to the underlying street network [41, 42]. This step is also context-dependent and it is motivated by the fact that we want to study how individuals interact with their built food environment, rather than how much they exercise in open spaces such as parks. In other words, when studying eating behaviours, we are more interested in “where” they are instead of “how active” they are.

Following the transformation into GPS trips, one needs to derive food outlet utilization and exposure. Exposure can be computed by listing all food venues that one has been exposed to, possibly with a weighing as a function of time spent. Utilization consists only of the set of food venues that one has interacted with, and one way to infer them is to use thresholds based on time [43]. It should be noted that computing both exposure and utilization requires knowing where the food locations are. Previous studies on the food environment conducted using GPS have either mapped all food locations and overlaid this map with GPS tracks or defined based on time spent at a location in the GPS track whether they ate at the food outlet [44, 45]. For either approach, it is necessary to include a map holding information (i.e., a GIS component) about the built environment; further technical background in combining GIS and GPS for health research can be found in [46–48] while a recent overview of gaps and solutions in obesity research is provided by [49]. In some countries, it is possible to request access to the location and the type of food outlets. For example, in England, type and location can be obtained via the Points of Interest data [50], which aggregates over 150 databases in the “eating and drinking” category and has an accuracy ranging from 81% to 100%.

#### 2.1.3. Investigating the Relationships between Food Exposure and Utilization Using Data Mining

Obtaining high quality datasets and performing the procedure outlined in the previous subsection will generate clean and usable data for an ABM. This data could be directly used to operationalize the agents. For example, each of the agents in the virtual world depicted by the ABM could be assigned a routine based on traces from the corresponding participant in the dataset. This is sufficient if one is interested in policies for the specific place where the dataset originates, but it cannot apply to the circumstance where one wishes to generalize the agents' navigation patterns. In this case, the agents' navigation cannot be simply assigned based on existing data: rather, the decision-making* rules* need to be extracted from the data. In other words, if one wishes to have agents that decide where to go instead of repeatedly following a preprogrammed path, then the data has to be mined.

The computational technique of data mining can be used to investigate the complex ways in which utilization of the food environment relates to exposure, while accounting for key sociodemographic factors (e.g., age, gender, and family structure). Data mining differs from statistical approaches such as regressions, which are traditionally used to investigate a variety of health behaviours [51, 52]. In data mining, the computer learns the relationships by being provided many cases. In our case, the goal is to understand food behaviour at the intersection of individual and environmental factors. Suggestions on what to collect in this context are provided by the dual-process view on the environment-behaviour relationship [53], stating that the environment has both a direct and indirect influence on behaviour. The indirect influence reflects the mediating role of behaviour-specific cognitions in the influence of the environment on behaviour. The direct influence reflects the automatic, unconscious influence of the environment on behaviour. Hence, one would seek to assemble data about the environment, sociodemographics, and cognitions. For example, the computer could be given what each individual was exposed to (via GPS traces) (there are issues of selective daily mobility bias. For example, one may go to a food store for various reason, and the GPS track will lead to that food store. We may erroneously infer that the person was “exposed” to the food store and finally chose to use it while it was the intention all along. Consequently, we do not recommend the sole use of GPS traces. Rather, they should be supplemented by travel diaries (which would clarify whether this food store was the initial goal) and surveys on food shopping behaviours. This may reduce but not eliminate issues of causalities arising in data mining. This is partly remedied at the ABM stage, where modellers complement the rules derived from the data with rules informed by the theory and apply calibration to identify appropriate rules), what they ended up using (e.g., via travel diaries), and what their beliefs and attitudes were (e.g., via surveys on food shopping behaviours), completed by the individual's age, gender, and family structure (note that we are primarily concerned about* supervised data mining*: we know the exposure and the outcome, and we provide these to the computer so that it can learn the relationships. It differs from* unsupervised data mining* in which we may not know the outcome and where the primary task is rather to identify similar individuals (e.g., clustering)). The computer then learns how exposure relates to utilization,* without having to assume* that this relationship takes a specific mathematical shape (e.g., a linear function). This advantage was highlighted by Dierker and colleagues, who noted that such data science techniques “allow for a data driven exploration of nonlinear relationships […] and have the potential to fit numerous interactions that cannot be handled as efficiently with either traditional regression techniques or other pattern centered methods” [54]. Being able to capture nonlinear relationships is particularly important for behaviours [55]: for example, “there may be common patterns of behaviour change within and across individuals that follow certain complex, nonlinear patterns” [56].

Several tools exist within data mining, depending on the task.* Classifiers* have previously been used in public health nutrition [57] and can be used to connect food exposure and utilization. Intuitively, a classifier is a function that assigns a label to a case (e.g., what type of food outlet was used by individuals) based on certain features (e.g., individuals' exposure and individual sociodemographic). There are many ways to build classifiers. For example, the same problem (e.g., identifying drinking behaviours) can be addressed using a variety of classifiers such as support vector machines [58], decision trees [59], or random forests [60]. While a discussion on which type of classifier is more appropriate to examine food exposure and utilization in one's dataset is beyond the scope of this review, it should be noted that the resources (i.e., computer space and time) required by a classifier can be a key criterion as one embarks into doing data science on large datasets. Indeed, the need for resources can grow faster than the increase in the size of the data [61]. Furthermore, GPS datasets consist of identifiable data, and ethics agreements may have been designed to limit where this data is stored. While researchers may work with ethics committees to allow encrypted data to be sent to a computing cluster, others may prefer to do all processing “in house” (which may be a condition of the ethics agreement).

GPS datasets can contain identifiable records: we can infer where individuals live and work. Data collection agreements often prohibit sharing identifiable records, even if sharing study data has been encouraged [62]. Consequently, sharing the data may either be a challenge or require a mix of agreements and anonymization procedures (e.g., segmenting the signals), which altogether limit the possibility of combining several studies to obtain a bigger picture of how food exposure relates to utilization. This can actually be mitigated by the use of classifiers, for three reasons. First, unlike the data used to build them, classifiers* can* be shared just in the same way as regression analyses. In other words, patterns (e.g., classifiers, regressions) are routinely communicated (e.g., through publications) while the raw data on which they were built may be kept private. Second, a classifier built for a dataset essentially provides a set of rules specifying how food exposure relates to utilization. Third, and most importantly, classifiers can be aggregated by “merging” their rules derived from local datasets in order to obtain a set of rules for the bigger picture [63]. This can intuitively be thought as doing a meta-analysis, where generalizability of the findings is improved by combining individual studies. It opens up the possibility, for example, to analyze GPS datasets in small geographical boundaries (e.g., city level or county level) and to pull together the analyses in order to understand the phenomenon at a bigger scale, while preserving the confidentiality of individual records. Mechanisms to merge classifiers can also account for the fact that classifiers derived from older datasets are less representative of current trends. This allows us to make use of the growing collection of GPS datasets while ensuring that the final product remains most representative of current trends.

It should be noted that, for a single dataset, one can derive a variety of classifiers either by selecting different methods to build classifiers, or by tuning the parameters of a given method [51]. In other words, for a single dataset, one can produce a variety of competing hypotheses to explain how food exposure relates to utilization. Selecting a hypothesis should take into account logical reasoning (when imaging how it translates to a public policy) and how well it fits the data. The fit can be computed using traditional measures in data mining (e.g., accuracy, sensitivity, and specificity) but also by running the different hypothesis in the ABM and observing the resulting behaviour of the agents.

### 2.2. Developing ABMs to Test Food Policies

#### 2.2.1. Creating the Rules of the Agents

Designing an ABM involves determining the decision-making rules of virtual people, or “agents.” Data mining can inform these rules by empirical research in two ways [51, 64]. First, it can show what factors should be involved in the rules (e.g., if it shows that repeated long exposure matters or that age matters more than they should be captured in the ABM). Second, it will also be essential for the calibration phase by giving the extent to which the ABM should be able to replicate observations from the GPS at baseline. For example, if data mining can explain 70% of the observations then it creates a benchmark against which to compare the ABM. (Note that the goal of an ABM is not necessarily to exceed this benchmark. For example, we previously developed an ABM which had the* same* accuracy as was obtained from data mining [51, 64]. The advantage of the ABM was that it had explicit hypotheses that could be communicated to policy makers, and modified if needed, in contrast with classifiers which may be difficult to comprehend (e.g., support vector machines creating vectors in a high dimensional space) and are not designed to be modified.)

The rules of the agents are often informed by both the data and field expertise. For example, the participants generating the GPS traces may not have disclosed their food preferences and health beliefs, but we know that these play a role in food choices and they are thus used in ABMs [14]. When having to supplement rules derived from the data with rules based on expertise, the rules should be grounded in a theoretical model of health behaviour. Numerous such models exist, including the multilevel theory of population health which emphasizes the role of habits [65], social ecological behaviour models that look at the importance of the food environment both physically and socially [66], and a variety of social-cognitive models that can include behavioural, normative, and control beliefs [67]. The existence of multiple models makes it difficult to formulate hypotheses (e.g., where to start, given possible differences between models). However, the flexibility of ABM to incorporate hypotheses is beneficial as it allows testing the potential impact of hypotheses derived from different models. This not only is relevant for public policy, but also fosters theory development in the area of food choices specifically and more broadly with regard to behavioural models in general.

#### 2.2.2. Parameters and Outputs

**Figure 1:**
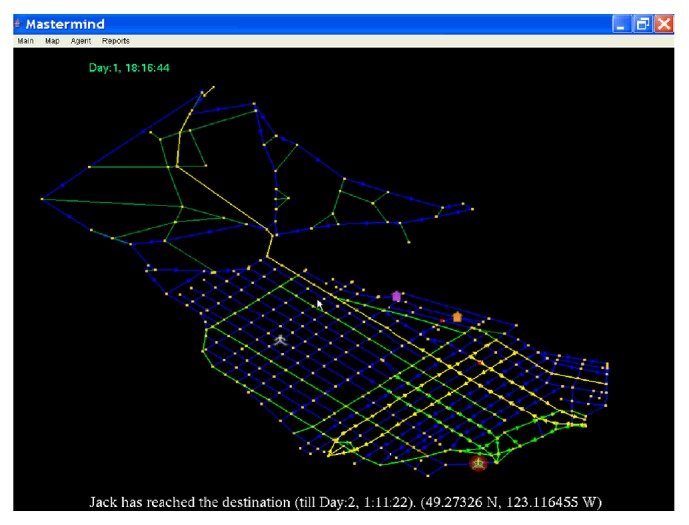
Simulation of human travel patterns in Mastermind [76] using the road network of downtown Vancouver, BC.

“What-if” scenarios, such as interventions that alter social and/or environmental sources of influence, can be tested using an ABM and produce estimates (e.g., impacts on diet and obesity). Supporting “what-if” scenarios is one of the main applications of modelling and simulation [68] and the design of such platforms has been well illustrated [9, 14, 69, 70] including in its application to policy portfolios for healthy food choices [14, 71].  Figure 1 provides as example a virtual platform simulating how individuals' behaviour depends on social and environmental influences, which could be changed to see the resulting impact in behaviours. A similar example can be found in [72] where a virtual platform simulates how individuals' physical activity behaviour could be changed.

In 2007, Ligmann-Zielinska and Jankowski wrote that “there exist only a handful of operational agent-based models. Most models are either highly abstract, missing the policy-driven context, and hence represent intellectually intriguing but practically insufficient tools, or are in an early stage of their operational development” [73]. While the number of ABMs has grown steadily since 2007, few can still be deemed operational. Thus, it is essential that future ABMs support a wide portfolio of policy interventions that at least includes what policymakers are interested in. This means including interventions ranging from urban planning to supporting individuals in making healthy choices [74, 75]. To support such wide range of interventions, an ABM should include parameters that would automatically adjust the built environment (e.g., maximum density of takeaways, minimum distance of takeaways from schools) as well as parameters that would directly affect the agents (e.g., preference for healthy foods).

In addition to ensuring that the ABM has a relevant range of tuneable policies through its input, close attention should be paid to the outputs of the model. Indeed, the ABM must provide a series of indicators to evaluate the impact of interventions. These indicators must be relevant to diet and adiposity, and they should also be supported by data. Indicators of adiposity include the Body Mass Index, or the waist-to-hip ratio, which captures the specific health risks of abdominal fat by taking into account the distribution of body fat. Indicators for diet can at least include utilization per type of food outlet, as this can be derived from the GPS dataset (cf. previous subsection) that informed the ABM. Indicators of diet quality would be an asset, although they are available in few GPS datasets. For example, the Fenland study includes plasma vitamin C level, which is considered a good biomarker of fruit and vegetable intake [77, 78] and thus provides information about overall diet quality.

When data on socioeconomic status is available, it can be used to explore the effects of different policies across socioeconomic strata [79]. ABM has been used only sparingly to assess food policies for low-income populations [15] while there is ample evidence that socioeconomic inequalities in diet contribute to inequalities in obesity and chronic disease risk. For example, a review of cross-sectional studies showed that higher-quality diets (e.g., nutrient-dense, with high variety and quantity of fruits and vegetables) are generally consumed by individuals with high socioeconomic status while lower-quality diets (e.g., energy dense) are consumed by individuals with lower socioeconomic status [80].

### 2.3. Using ABMs to Find Synergistic Combinations of Interventions That Promote Healthy Eating 

#### 2.3.1. Going beyond a Single Policy: Systems Thinking with Agent-Based Modelling

Food behaviours have been targeted by a variety of environmental-level and individual-level interventions. Recent illustrations can be found in cities such as Carlisle and Preston which have moved toward zoning restrictions for fast food outlets [74], while the UK Department of Health emphasized the potential of changing norms to promote behaviour change [75] (for a more complete overview of behaviour change methods we refer to [81]). As exemplified by these different approaches, there is a multiplicity of environments that influence food behaviours [82]: the physical environment (e.g., variety of food outlets), economic environment (e.g., cost of foods), political environment (e.g., food regulations), and sociocultural environment (e.g., beliefs and attitudes related to food). Developing interventions on food behaviours is thus complex as it addresses multiple sources of influence [83]. As ABMs can be used to inform the design and evaluation of complex interventions, it is particularly important that they reflect this multiplicity [6, 84].

When an ABM is built as explained in the previous subsection, its input parameters can be tuned so that a wide range of policies can be tested. An (overly simplistic) approach is to test policies one at a time, for example, by using one combination of parameter values to represent planning policies creating healthy buffers around schools, while another combination represents a communication campaign on attitudes toward healthy foods. There are two issues with such use of an ABM.

First, an intervention designed to change one element may have rippling consequences. For example, a high density of takeaways might lead to increased market competition through lowering prices and increasing portion sizes. Interventions limiting the density would decrease market competition, thus having potentially added benefits in lowering the prevalence of cheap unhealthy calories. At the same time, there may be initiatives to work with local businesses to promote healthier cooking practices. One such example was provided in a recent toolkit for London [85, p. 18]:

The East Midland's Eat Out Eat In Well scheme encouraged the Indian takeaways it was working with to use water rather than oil to keep food moist, and developed a new stock-point recipe using a dried spice mix rather than readymade mixes which were more expensive and had high oil content.

Both interventions could reduce the prevalence of cheap unhealthy calories, but through very different pathways. If “prevalence of cheap unhealthy calories” is one parameter in the ABM, then one may not be aware that it would be affected by both interventions or may not be able to adequately capture this interaction.

Second, and most importantly, research on cross-sectoral policy coherence has demonstrated that policies supporting healthy diets require coordination and capacity to work pragmatically across organizational, sectoral, and jurisdictional boundaries [86]. The various actors may have a range of capacity and work on different time scales, for example, because of their specific political cycles. Consequently, interventions that ultimately impact the same ABM parameter may not do so within the same time scale.

To adequately represent several interventions in the ABM, one would thus need to account for the rippling consequences and different time scales of the interventions. In other words, rather than imputing policies one at a time, there is a need for a systems thinking approach that accounts for multiple policies with interacting effects and delays. The advantages are clear: one may find combinations that benefit healthy eating much more than the sum of each separate intervention. However, this can be a technical challenge because the policies may need to be modelled in their own rights, thus adding an intermediate layer to interacting with the ABM.

#### 2.3.2. Adding an Intermediate Layer to Capture Policies in an ABM

**Figure 2:**
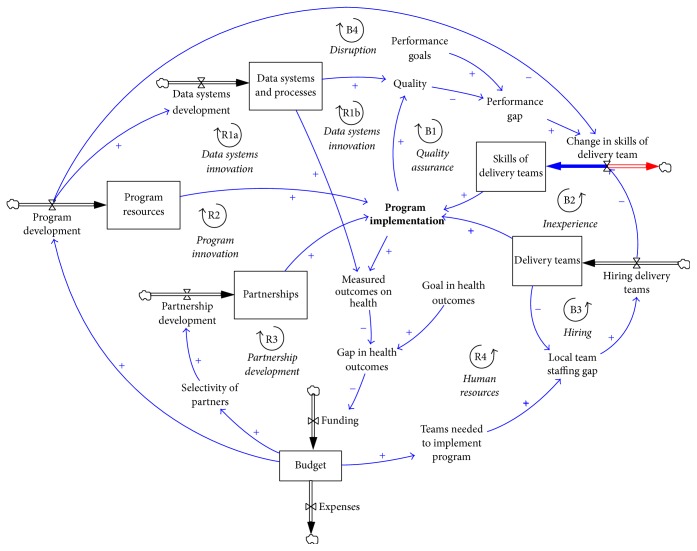
Viewing an intervention on overweight and obesity as a system using SD. This nonfinal model was sketched in the context of the “Mind, Exercise, Nutrition, Do It!” (MEND) program.* Reproduced with permission from Professor Peter S. Hovmand*.

Agent-based modelling is a quantitative* individual* technique that excels at representing how individuals interact with their environments and each other. Policies are going to affect this environment and/or individual interactions. At an abstract level, policies are composed of moving parts with interactions and delays. For example, the resource devoted to a program can interact with its implementation which, in turn, affects health outcomes (Figure 2). These two interactions can have different delays (e.g., insufficient resources can quickly jeopardize a program while health outcomes of obesity policies may take longer to manifest), and their relationship may be linear (e.g., the program may approximately scale with resources) or not (e.g., changes in weight have plateaus). ABM being a very flexible framework, these complex interactions can be represented within the model. However, significant efforts have been dedicated in the modelling community to develop System Dynamics (SD) models when the focus is on policies. SD is a quantitative* aggregate* technique (Figure 2), with an established track-record to “enhance our ability to understand the combination of strategies with potential for greatest impact” in the context of overweight and obesity [87]. Modellers are thus faced with a choice: they can develop the policy component of the model within the ABM, which would involve additional calibration and validation when done from scratch; alternatively, they can connect their ABM with validated SD models (there are many examples for the use of SD in obesity research, although more commonly to study changes in body weight over time at the individual [88–90] or population level [91, 92] than to examine potential impact of interventions [93–95]), thus creating a hybrid model.

Being able to integrate and reuse SD models would be particularly beneficial given that the modelling community devotes significant efforts to developing and validating such models (thus creating high quality “building blocks”). In addition, workshops have started training policymakers in using SD. For example, the Georgia Health Policy Center introducing SD to policymakers working on childhood obesity and, several months later, the General Assembly passed legislation on childhood obesity. As reported by the authors, “several attendees of our program commented that the level of conversation was different because of their experience with the model and impacted the passage of the legislation.” [94, p. 121] Such outcomes contribute to the reputation of SD with policymakers in the field of obesity, while training events may help them in expressing their mental models using the SD paradigm rather than the ABM paradigm. Consequently, considering the policy component as an additional SD layer in the model can contribute to a smoother development process with policymakers and ultimately in their interest to use the model.

While a broader discussion on hybrid models is beyond the scope of this paper which is primarily devoted to ABMs, we note that developing such models can be a nontrivial modelling endeavour. There are several reasons why modellers may choose to remain entirely within the ABM paradigm, other than the paradigm's ability to represent policies. Creating a hybrid adds complexities because the SD model needs to be connected to the ABM part: this requires identifying which factors act as outputs of the SD and input to the ABM and specifying exactly how they connect (which can be challenging). It may also require teams in which there are modellers familiar with ABM and with SD, thus adding logistical constraints.

## 3. Results and Discussion

This review is the first to articulate how techniques found in separate strands of literature can be brought together to develop the next generation of agent-based models for public policies about food behaviours. Our review was organized in three steps, following the process of model building. We first summarized how to acquire and analyze detailed datasets about individual mobility patterns, which results in better understanding of the complex relationships between food exposure and utilization. Then, building on that understanding and still using the data, we reviewed the key methodological elements in creating ABMs. Finally, we proposed to take a systems thinking perspective that views interventions as complex systems in their own rights such that their interactions and delays can be captured, and we synthesized how existing techniques can help achieve that goal.

While our three steps' approach can provide modellers with a practical plan for the development of future models for policy purposes, we did not aim at providing a complete overview of all steps involved in modelling and simulation. For example, we did not mention visualizations. This can be an important step, particularly when the model has to be usable and trusted by policymakers. As we previously discussed, interactive visualizations can be used at different stages in the development of models in chronic health [96]. The choice of a visualization will also involve the type of data expected at that stage and the nature of the interactions (i.e., who is the user group and what needs to be more salient for them in visual form). For example, policymakers may want to see how the space and/or the behaviours of people residing in it changes over time, under the effect of selected interventions. While one may replay the simulation as a movie, this may not allow policymakers and other observers to see temporal patterns or minute differences from one part of the simulation to another. Consequently, different visualizations have been proposed to deal with issues of change blindness in navigating simulation output over discrete spaces [97] (as produced by ABM). A review would be needed to examine the different ways in which visualizations can support the design, validation, and use of ABMs in policy research.

Validation will also require great attention from modellers, particularly as the validity of ABMs needs to be assessed before using them to make policy recommendations. Ideas and practical guidelines for the validation of ABMs were formulated by Bruch and Atwell. The models produced by our process would typically fall under the category of “Highly Realistic Models” and can be validated using the five steps' approach summarized by Bruch and Atwell. Some key considerations included in these steps are the specification and analysis of model uncertainty and the identification of measures of fit at multiple levels of granularity [98].

While visualization can play a role in all simulations, and validation is a requirement, an optional and specific aspect of policy-relevant ABMs is the possibility of improving the model using natural experiments. That is, once interventions suggested by the model start being implemented and their* actual* results are collected, they can be fed back into the ABM. Specifically, this would be an ongoing calibration process whereby the rules of the ABM are fine-tuned as the results of the interventions become available. Many natural experiments have been conducted when it comes to food behaviours, such as introducing food retailing in deprived communities [99] or varying the physical environment characteristics of dormitories and measuring changes in the students' weight and behaviours [100]. While we know how to manage and collect data regarding experiments, a standardization of their use to calibration policy-relevant ABMs remains to be explored in future work.

Articulating separate strands of the literature to develop the next generation of policy-relevant ABMs for food behaviours is an ambitious goal, particularly in light of the ABMs developed so far. This is an interdisciplinary endeavour and it may require sizeable teams composed, for example, of policymakers, health geographers, system modellers, computer scientists, and health psychologists. Navigating boundaries and managing disparate teams are common challenges with projects that are both technologically innovative and very applied; analyses of factors contributing to failure and successes of such projects can be found in [101]. Addressing these challenges is nonetheless essential to the creation of evidence-based policies.

Articulating together these three separate strands is applicable not only to the study of food behaviours, as explained here, but also for other behaviours such as active travel. Indeed, each strand has been applied independently for active travel: numerous GPS datasets have been analyzed to understand the relationship between the environment and active travel [48], while agent-based models have been developed that can assess responses to a change in environment [102]. There are thus opportunities to link these different pieces through a methodologically coherent framework that is presented in this review and ultimately supports decision-making.

We are aware that putting this process to practice requires a leap forward, but we believe that it is a long-term objective well-worth addressing to develop interventions that can effectively support healthy eating practices in the population. It could also lead to a paradigm shift. Instead of intervention developers trying to convince policy makers about their approach, this enables policy makers to guide intervention developers to come up with interventions with a potentially larger public health impact. This top-down approach might be common practice in some communities or even countries already, but giving policy makers tools to do this in an evidence-based is likely to be beneficial in comparison with current public health practice. This does not mean that policy makers will tell intervention developers* how* to develop interventions (after all, that is their expertise), but only what the focus of interventions might be or what type of interventions would be most beneficial to public health. Needless to say, these tools can also be used in a bottom-up approach allowing intervention developers to determine beforehand what focus or type of interventions has the highest potential.

## 4. Conclusions

Obesity being a complex condition, it is necessary to develop models that are mindful of this complexity. While current ABMs for food behaviours have numerous limitations and often ignore important components of the food environment, we reviewed and articulated how existing techniques can better capture how individuals navigate the food environment and develop public food policies.

## Figures and Tables

**Table 1:** Published studies having GPS data for over 100 participants.

Study	Number of participants	Age range	Country	Days	Measures BMI	Dietary measures
[27]	101	18–65	US	3	Yes	Survey on diet (FFQ) and food purchase (frequency)
[28]	119	18+	US	3	Yes	Survey on food shopping behaviours and dietary outcomes
[29]	175	13-14	UK	7	No	N/A
[30]	380	12–16	Canada	7	No	Dietary intake of healthy and unhealthy foods (FFQ): Harvard Youth/Adolescent Questionnaire (YAQ)
[31]	120	25+	US	7	No	Food-Frequency Questionnaire (FFQ)

## Abbreviations

ABM: Agent-based model
GPS: Global Positioning System
SD: System Dynamics.

## References

[1] Yang L., Colditz G. A. (2015). Prevalence of overweight and obesity in the United States, 2007–2012. *JAMA Internal Medicine*.

[2] Twells L. K., Gregory D. M., Reddigan J., Midodzi W. K. (2014). Current and predicted prevalence of obesity in Canada: a trend analysis. *Canadian Medical Association Journal*.

[3] Department of Health (2011). Healthy lives, healthy people: our strategy for public health in England. *White Paper*.

[4] Finkelstein E. A., Khavjou O. A., Thompson H. (2012). Obesity and severe obesity forecasts through 2030. *American Journal of Preventive Medicine*.

[5] Haby M. M., Markwick A., Peeters A., Shaw J., Vos T. (2012). Future predictions of body mass index and overweight prevalence in Australia, 2005–2025. *Health Promotion International*.

[6] Craig P., Dieppe P., Macintyre S., Mitchie S., Nazareth I., Petticrew M. (2008). Developing and evaluating complex interventions: the new Medical Research Council guidance. *The British Medical Journal*.

[7] Badham J. (2010). A compendium of modelling techniques. *Integration Insights*.

[8] Giabbanelli P. J., Alimadad A., Dabbaghian V., Finegood D. T. (2012). Modeling the influence of social networks and environment on energy balance and obesity. *Journal of Computational Science*.

[9] Giabbanelli P. J., Jackson P. J., Finegood D. T. (2014). Modelling the joint effect of social determinants and peers on obesity among Canadian adults. *Theories and Simulations of Complex Social Systems*.

[10] Shoham D. A. (2015). Advancing mechanistic understanding of social influence of obesity through personal networks. *Obesity*.

[11] Zhang J., Tong L., Lamberson P. J., Durazo-Arvizu R. A., Luke A., Shoham D. A. (2015). Leveraging social influence to address overweight and obesity using agent-based models: the role of adolescent social networks. *Social Science & Medicine*.

[12] Hammond R. A. (2010). Social influence and obesity. *Current Opinion in Endocrinology, Diabetes & Obesity*.

[13] Sleddens E. F. C., Kroeze W., Kohl L. F. M. (2015). Correlates of dietary behavior in adults: an umbrella review. *Nutrition Reviews*.

[14] Zhang D., Giabbanelli P. J., Arah O. A., Zimmerman F. J. (2014). Impact of different policies on unhealthy dietary behaviors in an urban adult population: an agent-based simulation model. *American Journal of Public Health*.

[15] Widener M. J., Metcalf S. S., Bar-Yam Y. (2013). Agent-based modeling of policies to improve urban food access for low-income populations. *Applied Geography*.

[16] Auchincloss A. H., Riolo R. L., Brown D. G., Cook J., Diez Roux A. V. (2011). An agent-based model of income inequalities in diet in the context of residential segregation. *American Journal of Preventive Medicine*.

[17] Kestens Y., Lebel A., Daniel M., Thériault M., Pampalon R. (2010). Using experienced activity spaces to measure foodscape exposure. *Health and Place*.

[18] Fleischhacker S. E., Evenson K. R., Rodriguez D. A., Ammerman A. S. (2011). A systematic review of fast food access studies. *Obesity Reviews*.

[19] Caspi C. E., Sorensen G., Subramanian S. V., Kawachi I. (2012). The local food environment and diet: a systematic review. *Health & Place*.

[20] Burgoine T., Forouhi N. G., Griffin S. J., Wareham N. J., Monsivais P. (2014). Associations between exposure to takeaway food outlets, takeaway food consumption, and body weight in Cambridgeshire, UK: population based, cross sectional study. *The British Medical Journal*.

[21] Hillier A., Cannuscio C. C., Karpyn A., McLaughlin J., Chilton M., Glanz K. (2011). How far do low-income parents travel to shop for food? Empirical evidence from two urban neighborhoods. *Urban Geography*.

[22] Cetateanu A., Jones A. (2016). How can GPS technology help us better understand exposure to the food environment? A systematic review. *SSM—Population Health*.

[23] Li Y., Berenson J., Gutiérrez A., Pagán J. A. (2016). Leveraging the food environment in obesity prevention: the promise of systems science and agent-based modeling. *Current Nutrition Reports*.

[24] Levy D. T., Mabry P. L., Wang Y. C. (2011). Simulation models of obesity: a review of the literature and implications for research and policy. *Obesity Reviews*.

[25] Shoham D. A., Hammond R., Rahmandad H., Wang Y., Hovmand P. (2015). Modeling social norms and social influence on obesity. *Current Epidemiology Reports*.

[26] Mabry P. L., Bures R. M. (2014). Systems science for obesity-related research questions: an introduction to the theme issue. *American Journal of Public Health*.

[27] Christian W. J. (2012). Using geospatial technologies to explore activity-based retail food environments. *Spatial and Spatio-Temporal Epidemiology*.

[28] Gustafson A., Christian J. W., Lewis S., Moore K., Jilcott S. (2013). Food venue choice, consumer food environment, but not food venue availability within daily travel patterns are associated with dietary intake among adults, Lexington Kentucky 2011. *Nutrition Journal*.

[29] Harrison F., Burgoine T., Corder K., van Sluijs E. M. F., Jones A. (2014). How well do modelled routes to school record the environments children are exposed to? A cross-sectional comparison of GIS-modelled and GPS-measured routes to school. *International Journal of Health Geographics*.

[30] Shearer C., Rainham D., Blanchard C., Dummer T., Lyons R., Kirk S. (2015). Measuring food availability and accessibility among adolescents: moving beyond the neighbourhood boundary. *Social Science and Medicine*.

[31] Zenk S. N., Schulz A. J., Matthews S. A. (2011). Activity space environment and dietary and physical activity behaviors: A Pilot Study. *Health and Place*.

[32] Maglio P. P., Mabry P. L. (2011). Agent-based models and systems science approaches to public health. *American Journal of Preventive Medicine*.

[33] Nianogo R. A., Arah O. A. (2015). Agent-based modeling of noncommunicable diseases: a systematic review. *American Journal of Public Health*.

[34] Gasevic D., Matteson C. L., Vajihollahi M., Acheson M. A., Lear S. A., Finegood D. T. (2010). Data gaps in the development of agent-based models of physical activity in the built environment. *Obesity Reviews*.

[35] Yang Y., Diez Roux A. V., Auchincloss A. H., Rodriguez D. A., Brown D. G. (2011). A spatial agent-based model for the simulation of adults' daily walking within a city. *American Journal of Preventive Medicine*.

[36] Yang Y., Diez Roux A. V., Auchincloss A. H., Rodriguez D. A., Brown D. G. (2012). Exploring walking differences by socioeconomic status using a spatial agent-based model. *Health and Place*.

[37] Salathé M., Kazandjieva M., Lee J. W., Levis P., Feldman M. W., Jones J. H. (2010). A high-resolution human contact network for infectious disease transmission. *Proceedings of the National Academy of Sciences of the United States of America*.

[38] Petrenko A., Bell S., Stanley K., Qian W., Sizo Q., Knowles D. (2013). Human spatial behavior, sensor informatics, and disaggregate data. *Spatial Information Theory. COSIT 2013*.

[39] Transportation Research Board of the National Academies (2014). Applying GPS data to understand travel behavior. Volume I: background, methods, and tests.

[40] Krumm J. The geographic context browser.

[41] Scott C. Improved GPS positioning for motor vehicles through map matching.

[42] Zhang X., Wang Q., Wan D. The relationship among vehicle positioning performance, map quality, and sensitivities and feasibilities of map-matching algorithms.

[43] Giabbanelli P. J., Burgoine T., Monsivais P., Woodcock J. Using big data to develop individual-centric models of food behaviours.

[44] Thornton L. E., Pearce J. R., Kavanagh A. M. (2011). Using Geographic Information Systems (GIS) to assess the role of the built environment in influencing obesity: a glossary. *International Journal of Behavioral Nutrition and Physical Activity*.

[45] Chaix B., Méline J., Duncan S. (2013). GPS tracking in neighborhood and health studies: a step forward for environmental exposure assessment, A step backward for causal inference?. *Health and Place*.

[46] Jankowska M. M., Schipperijn J., Kerr J. (2015). A framework for using GPS data in physical activity and sedentary behavior studies. *Exercise and Sport Sciences Reviews*.

[47] Kerr J., Duncan S., Schipperjin J. (2011). Using global positioning systems in health research: a practical approach to data collection and processing. *American Journal of Preventive Medicine*.

[48] Schipperijn J., Kerr J., Duncan S., Madsen T., Klinker C. D., Troelsen J. (2014). Dynamic accuracy of GPS receivers for use in health research: a novel method to assess GPS accuracy in real-world settings. *Frontiers in Public Health*.

[49] James P., Jankowska M., Marx C. (2016). ‘Spatial Energetics’: integrating data from GPS, accelerometry, and GIS to address obesity and inactivity. *American Journal of Preventive Medicine*.

[50] Fraser L. K., Clarke G. P., Cade J. E., Edwards K. L. (2012). Fast food and obesity: a spatial analysis in a large United Kingdom population of children aged 13–15. *American Journal of Preventive Medicine*.

[51] Crutzen R., Giabbanelli P. (2014). Using classifiers to identify binge drinkers based on drinking motives. *Substance Use & Misuse*.

[52] Crutzen R., Giabbanelli P. J., Jander A., Mercken L., de Vries H. (2015). Identifying binge drinkers based on parenting dimensions and alcohol-specific parenting practices: building classifiers on adolescent-parent paired data. *BMC Public Health*.

[53] Kremers S. P. J., de Bruijn G.-J., Visscher T. L. S., van Mechelen W., de Vries N. K., Brug J. (2006). Environmental influences on energy balance-related behaviors: a dual-process view. *International Journal of Behavioral Nutrition and Physical Activity*.

[54] Dierker L., Rose J., Tan X., Li R. (2010). Uncovering multiple pathways to substance use: a comparison of methods for identifying population subgroups. *Journal of Primary Prevention*.

[55] Miller W. R. (2004). The phenomenon of quantum change. *Journal of Clinical Psychology*.

[56] Resnicow K., Vaughan R. (2006). A chaotic view of behavior change: a quantum leap for health promotion. *International Journal of Behavioral Nutrition and Physical Activity*.

[57] Giabbanelli P. J., Adams J. (2016). Identifying small groups of foods that can predict achievement of key dietary recommendation: data mining of the UK National Diet and Nutrition Survey, 2008–12. *Public Health Nutrition*.

[58] Zuba M., Gilbert J., Wu Y., Bi J., Tennen H., Armeli S. 1-Norm support vector machine for college drinking risk factor identification.

[59] McKenzie D. P., McFarlane A. C., Creamer M. (2006). Hazardous or harmful alcohol use in Royal Australian Navy veterans of the 1991 Gulf War: identification of high risk subgroups. *Addictive Behaviors*.

[60] Hillemacher T., Frieling H., Wilhelm J. (2012). Indicators for elevated risk factors for alcohol-withdrawal seizures: an analysis using a random forest algorithm. *Journal of Neural Transmission*.

[61] Drucker H., Wu D., Vapnik V. N. (1999). Support vector machines for spam categorization. *IEEE Transactions on Neural Networks*.

[62] Crutzen R., Peters G.-J. Y., Abraham C. (2012). What about trialists sharing other study materials?. *British Medical Journal*.

[63] Giabbanelli P. J., Peters J. (2015). An algebraic approach to combining classifiers. *Procedia Computer Science*.

[64] Giabbanelli P. J., Crutzen R. (2013). An agent-based social network model of binge drinking among Dutch adults. *Journal of Artificial Societies and Social Simulation*.

[65] Zimmerman F. J. (2013). Habit, custom, and power: a multi-level theory of population health. *Social Science and Medicine*.

[66] Booth S. L., Sallis J. F., Ritenbaugh C. (2001). Environmental and societal factors affect food choice and physical activity: rationale, influences, and leverage points. *Nutrition Reviews*.

[67] Fishbein M., Ajzen I. (2010). *Predicting and Changing Behavior: The Reasoned Action Approach*.

[68] Epstein J. M. (2008). Why model?. *Journal of Artificial Societies and Social Simulation*.

[69] Giabbanelli P. J. (2014). Modelling the spatial and social dynamics of insurgency. *Security Informatics*.

[70] Woodcock J., Givoni M., Morgan A. S. (2013). Health impact modelling of active travel visions for England and Wales using an Integrated Transport and Health Impact Modelling Tool (ITHIM). *PLoS ONE*.

[71] Struben J., Chan D., Dubé L. (2014). Policy insights from the nutritional food market transformation model: the case of obesity prevention. *Annals of the New York Academy of Sciences*.

[72] Ligmann-Zielinska A., Grady S. C., McWhorter J., Neal Z. P. (2006). The impact of urban form on weight loss. *Handbook of Applied System Science*.

[73] Ligmann-Zielinska A., Jankowski P. (2007). Agent-based models as laboratories for spatially explicit planning policies. *Environment and Planning B: Planning and Design*.

[74] UK Healthy Cities Network (2011). *Information Booklet*.

[75] Local Government Association (2013). *Changing Behaviours in Public Health: To Nudge Or to Shove?*.

[76] Brantingham P., Glasser U., Jackson P., Vajihollahi M. (2009). Modeling criminal activity in urban landscapes. *Mathematical Methods in Counterterrorism*.

[77] Harding A.-H., Wareham N. J., Bingham S. A. (2008). Plasma vitamin C level, fruit and vegetable consumption, and the risk of new-onset type 2 diabetes mellitus. *JAMA Internal Medicine*.

[78] Bates C. J., Thurnham D. I., Bingham S. A., Margetts B. M., Nelson M., Margetts B. M., Nelson M. (1997). Biochemical markets of nutrient intake. *Design Concepts in Nutritional Epidemiology*.

[79] White M., Adams J., Heywood P. (2009). How and why do interventions that increase health overall widden inequalities within populations. *Social Inequality and Public Health*.

[80] Darmon N., Drewnowski A. (2008). Does social class predict diet quality?. *American Journal of Clinical Nutrition*.

[81] Kok G., Gottlieb N. H., Peters G.-J. Y. (2016). A taxonomy of behaviour change methods: an Intervention Mapping approach. *Health Psychology Review*.

[82] Swinburn B., Egger G., Raza F. (1999). Dissecting obesogenic environments: the development and application of a framework for identifying and prioritizing environmental interventions for obesity. *Preventive Medicine*.

[83] Johnson-Askew W. L., Fisher R. A., Yaroch A. L. (2009). Decision making in eating behavior: state of the science and recommendations for future research. *Annals of Behavioral Medicine*.

[84] Chalabi Z., Lorenc T. (2013). Using agent-based models to inform evaluation of complex interventions: examples from the built environment. *Preventive Medicine*.

[85] Bagwell S., O'Keefe E., Doff S., Kumarappan L. Encouraging healthier takeaways in low-income communities: tools to support those working to encourage healthier catering amongst fast food takeaways.

[86] Dubé L., Addy N. A., Blouin C., Drager N. (2014). From policy coherence to 21st century convergence: a whole-of-society paradigm of human and economic development. *Annals of the New York Academy of Sciences*.

[87] Frerichs L. M., Araz O. M., Huang T. T.-K. (2013). Modeling social transmission dynamics of unhealthy behaviors for evaluating prevention and treatment interventions on childhood obesity. *PLoS ONE*.

[88] Rahmandad H. (2014). Human growth and body weight dynamics: an integrative systems model. *PLoS ONE*.

[89] Hamid T. K. A. (2009). *Thinking in Circles about Obesity: Applying Systems Thinking to Weight Management*.

[90] Chow C. C., Hall K. D. (2008). The dynamics of human body weight change. *PLoS Computational Biology*.

[91] Fallah-Fini S., Rahmandad H., Huang T. T.-K., Bures R. M., Glass T. A. (2014). Modeling US adult obesity trends: a system dynamics model for estimating energy imbalance gap. *American Journal of Public Health*.

[92] Homer J. B., Hirsch G. B. (2006). System dynamics modeling for public health: background and opportunities. *American Journal of Public Health*.

[93] Giabbanelli P. J., Torsney-Weir T., Finegood D. T. (2011). Building a system dynamics model of individual energy balance related behaviour. *Canadian Journal of Diabetes*.

[94] Minyard K. J., Ferencik R., Phillips M. A., Soderquist C. (2014). Using systems thinking in state health policymaking: an educational initiative. *Health Systems*.

[95] Verigin T., Giabbanelli P. J., Davidsen P. I. Supporting a systems approach to healthy weight interventions in British Columbia by modelling weight and well-being.

[96] Giabbanelli P. J., Jackson P. J. (2015). Using visual analytics to support the integration of expert knowledge in the design of medical models and simulations. *Procedia Computer Science*.

[97] Giabbanelli P. J., Babu G. J., Baniukiewicz M. A novel visualization environment to support modelers in analyzing data generated by cellular automata.

[98] Bruch E., Atwell J. (2015). Agent-based models in empirical social research. *Sociological Methods & Research*.

[99] Cummins S., Petticrew M., Higgins C., Findlay A., Sparks L. (2005). Large scale food retailing as an intervention for diet and health: quasi-experimental evaluation of a natural experiment. *Journal of Epidemiology and Community Health*.

[100] Kapinos K. A., Yakusheva O. (2011). Environmental influences on young adult weight gain: evidence from a natural experiment. *Journal of Adolescent Health*.

[101] Jackson P., Sixsmith J., Mihailidis A., Sixsmith A., Geissbühler A., Demongeot J., Mokhtari M., Abdulrazak B., Aloulou H. (2015). Perspectives on collaboration in technology innovation for ageing. *Inclusive Smart Cities and e-Health: 13th International Conference on Smart Homes and Health Telematics, ICOST 2015, Geneva, Switzerland, June 10–12, 2015, Proceedings*.

[102] Abbas S. M. A., Chalabi Z., Aldred R., Woodcock J. Use of an agent-based model to explore urban transitions in commuter cycling.

